# One-Step Electrochemical Preparation of Multilayer Graphene Functionalized with Nitrogen

**DOI:** 10.1186/s11671-017-1957-4

**Published:** 2017-03-09

**Authors:** Olena Ustavytska, Yaroslav Kurys, Vyacheslav Koshechko, Vitaly Pokhodenko

**Affiliations:** 0000 0004 0385 8977 grid.418751.e L.V. Pysarzhevsky Institute of Physical Chemistry of NAS of Ukraine, Prospect Nauky, 31, Kyiv, 03028 Ukraine

**Keywords:** Nitrogen-doped graphene, Electrochemical exfoliation, Sodium azide, Electrocatalysis, Electroanalysis

## Abstract

A new environmentally friendly one-step method for producing multilayer (preferably 7–9 layers) nitrogen-doped graphene (N-MLG) with a slight amount of oxygen-containing defects was developed. The approach is based on the electrochemical exfoliation of graphite electrode in the presence of azide ions under the conditions of electrolysis with pulse changing of the electrode polarization potential. It was found that usage of azide anions lead not only to the exfoliation of graphite but also to the simultaneous functionalization of graphene sheets by nitrogen atoms (as a result of electrochemical decomposition of azide anions with ammonia evolution). Composition, morphology, structure, and electrochemical properties of N-MLG were characterized by C,H,N analysis, transmission electron microscopy, atomic force microscopy, FTIR, UV–Vis, and Raman spectroscopy, as well as cyclic voltammetry. The perspective of using N-MLG as oxygen reduction reaction electrocatalyst and for the electrochemical analysis of biomarkers (dopamine, ascorbic acid, and uric acid) in their mixtures was shown.

## Background

Graphene, as a 2D carbon nanomaterial in which sp^2^-hybridized carbon atoms aligned in a honeycomb lattice, has attracted tremendous research interest due to its excellent electrical conductivity, high specific surface area, unique physical characteristics, mechanical properties, and chemical stability [1–3]. Functionalization of single-layer or multilayer graphenes by doping with different heteroatoms, in particular by nitrogen, allows controllable change electronic structure and, consequently, desirable properties of corresponding 2D materials [4]. It opens up new opportunities for creating of multifunctional nanostructured carbon materials, dispersions, and hybrid composites, used in catalysis, power engineering, biomedicine, “smart” materials and systems, etc.

Nitrogen-doped graphene (N-graphene) can be prepared via direct incorporation of nitrogen atoms into graphene by means of, for example, chemical vapor deposition, arc discharge, and solvothermal processes [5–7] or by N-doping of initially prepared graphene oxide (graphene) under thermal, plasma, electrochemical, etc. post-treatment [8–11]. In the first case, the main disadvantages are the harsh reaction conditions, sufficiently long duration, and high cost of processes due to using of specific equipment and necessity of strict implementation of manufacturing operations. Major drawbacks of the second approach are the multistaging of process and the using of environmentally hazardous reagents.

The electrochemical exfoliation of graphite is a promising approach to produce graphene and graphene-related materials due to its easy, fast, and environmentally friendly nature [3]. Recently, one-step production of multilayer N-graphene by electrochemical exfoliation of graphite electrode in aqueous electrolytes based on protic ionic liquid (ethylammonium nitrate) [12], ammonium nitrate [13], or (NH_4_)_2_SO_4_ and NH_4_OH [14] were reported. However, despite the advantages of the proposed approaches, their wide use is limited by the high cost of ionic liquid [12], high content of unwanted oxygen-containing defects in N-graphene [12–14], environmentally adverse concentrated solution of ammonia, using a sufficiently high potential, and a prolonged ultrasonic treatment [14].

Previously, we have shown the possibility of the formation of multilayer graphene (MLG) with slight amounts of oxygen-containing defects by means of exfoliation of graphite electrode in presence of benzoate anions in a pulse mode of electrolysis [15]. It is supposed that usage of azide anions instead of carboxylate anions can lead not only to the exfoliation of graphite but also to the simultaneous functionalization of graphene sheets by nitrogen atoms (as a result electrochemical decomposition of azide anions).

Considering the above, the purpose of this study was to establish the possibility of electrochemical one-step production of N-graphene with slight amounts of oxygen-containing defects via exfoliation of graphite in an aqueous solution of sodium azide in a pulse mode of electrolysis without using concentrated ammonia solution, expensive ionic liquids, and high potentials, as well as clarification of its electrocatalytic activity in the oxygen reduction and oxidation of such biomarkers as ascorbic acid (AA), dopamine (DA), and uric acid (UA).

## Methods

### Chemicals and Materials

High-purity graphite rods (Alfa Aesar, 99.9995%), gasses (Ar and O_2_), and commercially available chemicals (analytical grade)—NaN_3_, KCl, H_2_SO_4_, ascorbic acid, dopamine, and uric acid—were used as supplied without additional purification. The distilled water was used for electrolyte preparation. The graphene oxide (GO), used in the study for comparison, was obtained via liquid phase exfoliation of graphite oxide, synthesized by the modified Hummers method [16].

### Apparatus

Electrochemical studies were carried out via computer complex based on potentiostat PI-50-1.1 using a three-electrode undivided cell (working electrode—glassy carbon (GC) disk with visible surface area of 0.03 cm^2^; the auxiliary electrode—platinum mesh; reference electrode—Ag/AgCl, 3 M KCl). In order to modify electrode, 2 μL of aqueous or alcoholic dispersion (1 mg/mL) of the corresponding graphene material was dropped onto its surface, followed by drying on air. TEM images were recorded using a transmission electron microscope TEM125K (Selmi) with an accelerating voltage 100 kV (samples were deposited onto copper grids coated with amorphous carbon film). Atomic force microscopy (AFM) of thin film graphene samples on the surface of silicon wafers coated with silicon nitride (Agar Scientific) was performed on a Nanoscope IIIa Dimension 3000TM (Digital) instrument. FTIR spectra were taken on Fourier transform infrared spectroscope SPECTRUM ONE (PerkinElmer); samples were prepared as tablets with KBr. UV–Vis spectra of dispersions were registered via UV–visible spectrometer 4802 (Unico). Raman spectra were obtained with a triple spectrometer (Horiba Jobin-Yvon T64000, Ar–Kr laser, *λ* = 514 nm); samples were deposited onto silicon templates. C,H,N-elemental analysis was performed on Carlo Erba 1106 elemental analyzer (Carlo Erba, Italy) based on modification of the classical Pregl and Dumas method (combustion temperature of 1030 °C, atmosphere of oxygen) using 0.5–1.0 mg of sample per analysis. The oxygen content in the samples was evaluated by difference between the total weight of the samples and content C,H,N in them (on the basis of C,H,N analysis data).

### Synthesis Procedure

**Fig. 1:**
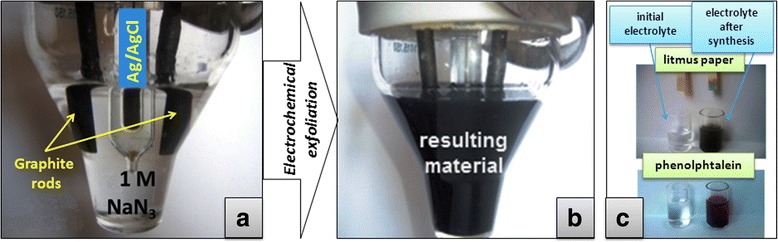
Color (**a**, **b**) and pH (**c**) differences of electrolyte before (**a**, **c**) and after (**b**, **c**) electrochemical exfoliation of graphite electrode in 1 M NaN_3_

Electrochemical exfoliation of graphite was carried out in undivided cell (working and auxiliary electrodes—graphite rods; reference electrode—Ag/AgCl) (Fig. 1a) using a potentiostat PI-50-1.1. Synthesis procedure was carried out in a pulse mode of electrolysis (analogous to [15]—polarization of electrode by +4 and 0 V (both throughout 50 s) with multiple changing of polarization potential (during, ordinarily, 20 h). One molar aqueous solution of NaN_3_ was used as electrolyte. Dispersion of obtained graphene material was filtered through a membrane filter with a pore diameter 0.2 μm (SUPELCO°), rinsed with water, and dried in oven at 60 °C. If there was necessity, the dried precipitate was transferred to appropriate solvent (for example, water), where regeneration of material dispersion was occurred by using ultrasound treatment for 2 min in the ultrasonic washing bath (Selmi). For comparison, the synthesis was carried out using other electrolyte concentrations: 0.1 and 2 M.

## Results and Discussion

During electrolysis, it was observed the gradual change of electrolyte color from colorless to gray and then to dark gray (Fig. 1b), which indicates the transition of graphene sheets in the electrolyte volume as a result of exfoliation of the graphite electrode. It is believed that during positive electrode polarization, the intercalation of azide anions (N_3_^−^) into graphite interlayer space followed by its partial anodic decomposition, N_3_^−^ → 3/2 N_2_ + e^−^ [17], take place. In case of application to electrode of potential 0 V, the deintercalation of N_3_^−^ occurs and also its partial decomposition: N_3_^−^ + 3H_2_O + 2e^−^ → N_2_ + NH_3_ + 3OH^−^ [18]. Multiple repetition of anion intercalation/deintercalation cycles into graphite interlayer space as well as N_2_, NH_3_, and O_2_ evolution during electrolysis provide separation of graphene layers, forming multilayer packages of graphene, doped with nitrogen, that are passing to electrolyte volume. At the same time, ammonia, evolved as a result of cathodic decomposition of N_3_^−^, acts as a nitrogen source for the in situ graphene doping, while using of low potentials and nitrogen evolution as a result of electrochemical process promotes low number of oxygen-containing defects in N-graphene. Furthermore, over time pH of the electrolyte was changed from 7 to 11–12 (Fig. 1c), confirming the formation of ammonia and hydroxyl anions—as a result of the partial electrochemical decomposition of azide ions, that is evidence in favor assumption, made above, about the mechanism of the process under the used conditions.

**Fig. 2:**
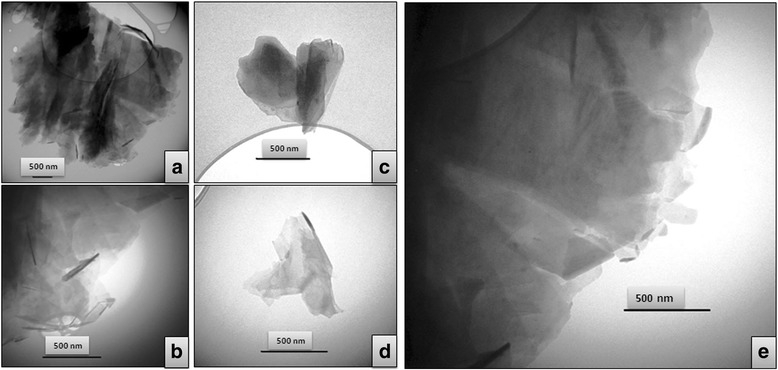
**a**–**e** Typical TEM images of N-MLG

On the TEM images of obtained material (Fig. 2), multiple-layered lamellar structures, which consist of graphene sheets with lateral size that ranges from several hundred nanometers (Fig. 2c, d) to several microns (Fig. 2a, b, e), are observed. Thus, the TEM data indicate that the preferred product of the electrochemical exfoliation of graphite in aqueous NaN_3_ is multilayer graphene (N-MLG). It should be noted the appearance of so-called Moire contrast on some TEM micrographs of the N-MLG particles (Fig. 2e), which can be caused by a slight misorientation (displacement relative to each other) of graphene layers in a multilayer package [19, 20]. A similar effect was observed earlier for electrochemically obtained MLG by using benzoate anions as the electrolyte [15].

**Fig. 3:**
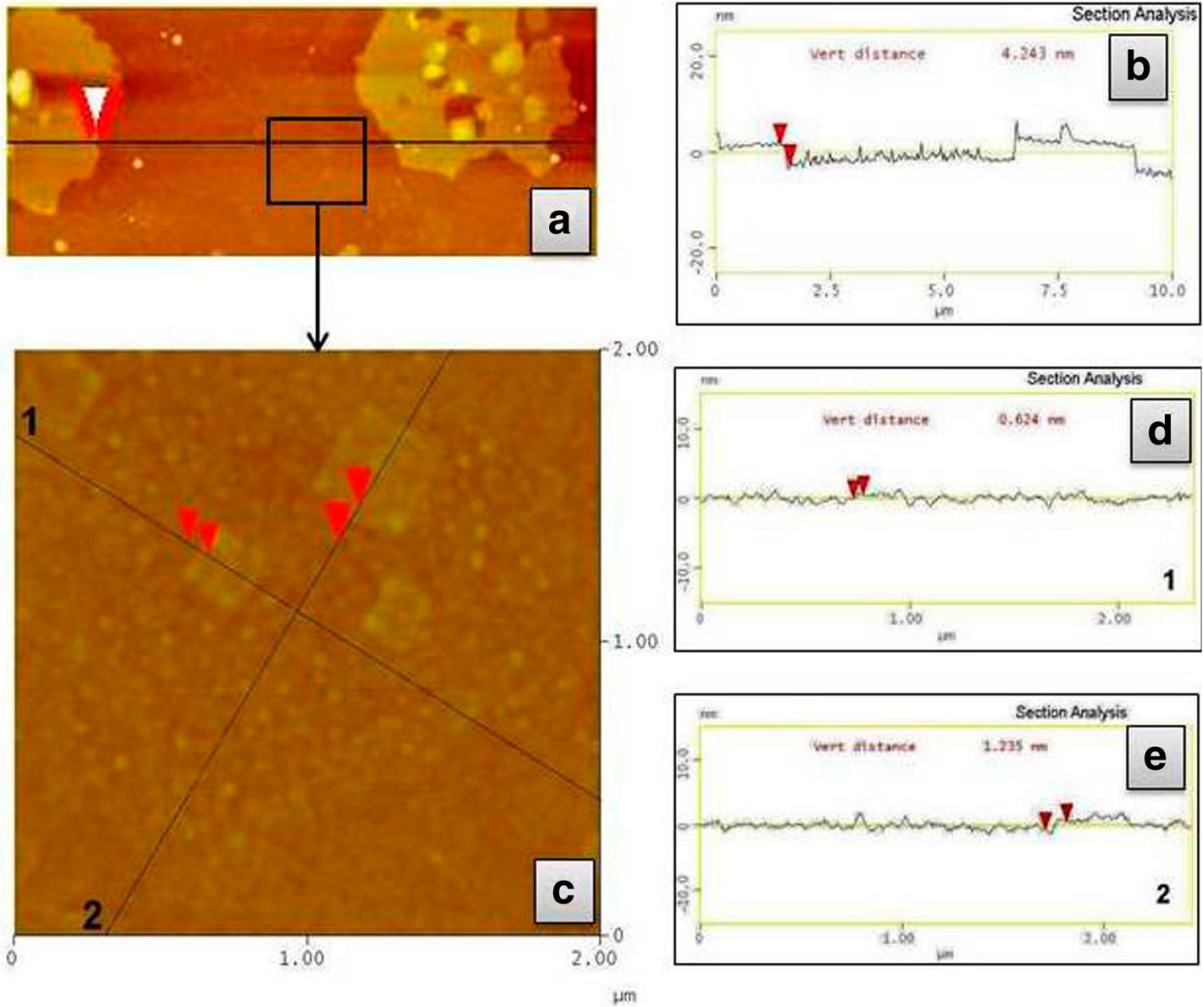
AFM images (**a**, **c**) and corresponding cross-sectional profiles of N-MLG (**b**, **d**, **e**)

As a result of electrochemical exfoliation of graphite electrode in aqueous electrolyte based on NaN_3_, mostly dispersions of multilayer N-graphene are obtained. This fact is evidenced by AFM data (Fig. 3). The thickness of N-MLG particles from diluted ethanol dispersion (determined based on the corresponding cross-sectional profiles on Fig. 3b, d, e) reaches predominantly 4.2–5.4 nm. Also, it should be noted that values of thicknesses of measured particles are aliquot to 0.6; at the same time, the smallest thickness of the particle, which we managed to register, is also 0.6 nm. Hence, it can be assumed that 0.6 nm is the thickness of a monolayer of obtained material and, consequently, rest particles of N-MLG in dispersion are packages containing up to 7–9 monolayers. Apart from multilayer N-MLG particles, the particles with the thickness of 0.6–1.8 nm and lateral size of 200–500 nm are also exist in the dispersion; they correspond to single- or several-layer (2–3 single layers) N-graphene. The lateral size of N-MLG particles, determined based on AFM data (Fig. 3), is agreed with TEM data.

**Fig. 4:**
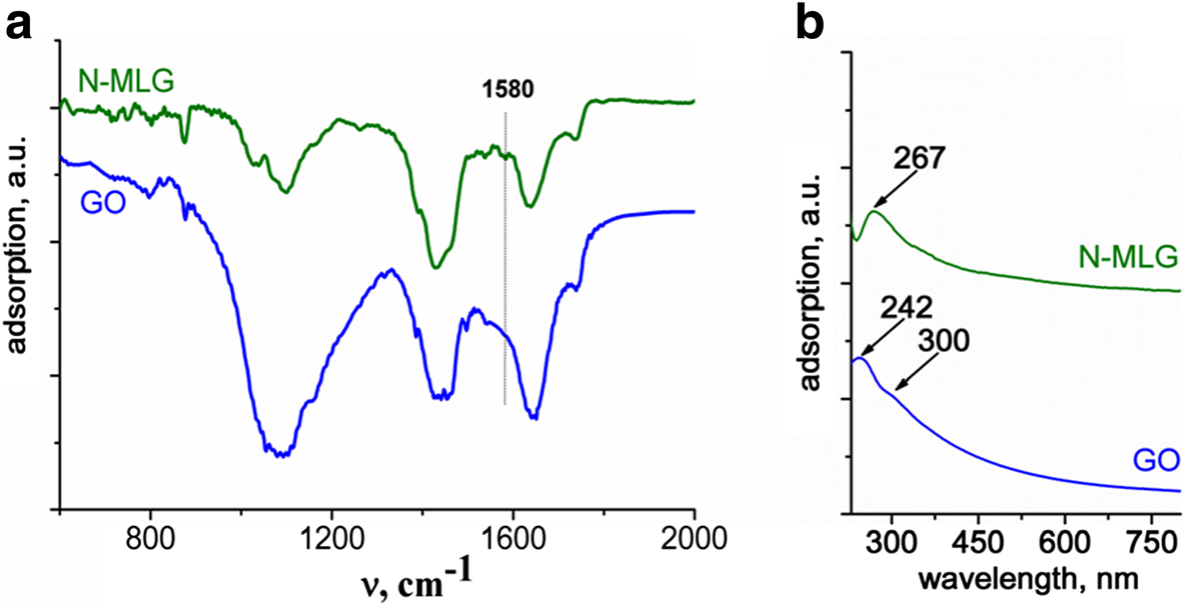
FTIR (**a**) and UV–Vis (**b**) spectra of N-MLG and GO

Figure 4a shows the FTIR spectra of N-MLG in comparison with graphene oxide (GO), obtained via modified Hummers method [16]. Characteristic bands, related to bond vibrations in oxygen-containing fragments [21–23]—C=O in carboxyl or carbonyl (1740 cm^−1^), C–O in carboxyl (1460 cm^−1^), and C–O in epoxy and alkoxy (1100 cm^−1^), are observed in both spectra. It should be noted much smaller intensity of bands caused by vibrations in oxygen-containing groups in N-MLG spectrum if compare with spectrum of GO, which indicates on significantly lower content of such groups in obtained MLG doped with nitrogen. The unambiguous interpretation of intense band at 1629 cm^−1^, which present in spectra on Fig. 4a, is difficult because of the possibility of its assigning to a deformation vibrations of adsorbed water molecules and to fluctuations in the C=C bonds in unoxidized sp^2^-C clusters of graphene [21–23]. It is important to note the presence of a band at 1580 cm^−1﻿^ in FTIR spectrum ﻿of N-MLG, unlike GO, related to C–N stretching vibrations [24], which confirms formation of MLG, doped with nitrogen.

Partially oxidized state of obtained material and functionalization of it by nitrogen were also confirmed by C,H,N-analysis data. Thus, calculated nitrogen content in N-MLG was about 0.6% and atomic ratio C/O ~17. Such C/O ratio indicates that oxygen-containing groups although are present in obtained N-MLG, but their content is much less than for example in chemically or thermally reduced GO, where C/O ~8–11 [25]. It is important to note that increase of electrolyte concentration from 0.1 to 2 M leads to symbate increase of nitrogen content in resulting material from 0.2 to 0.9%, which opens up the perspectives of controlling the nitrogen content in multilayer graphene, obtained by proposed method.

The presence of band with maximum absorption at 267 nm in UV–Vis spectrum of N-MLG dispersion in ethanol (Fig. 4b), which corresponds to the so-called van Hove singularity in the graphene density of states [26], evidenced in support a slight oxidation of the obtained N-functionalized graphene. At the same time, UV–Vis spectrum of highly oxidized GO dispersion (Fig. 4b) differs from investigated UV–Vis spectrum of N-MLG: maximum of adsorption is observed at 242 nm as well as a shoulder at about 300 nm associated with the nπ*-junction with the participation of unshared electron pairs of the oxygen atoms in oxygen-containing groups [26].

**Fig. 5:**
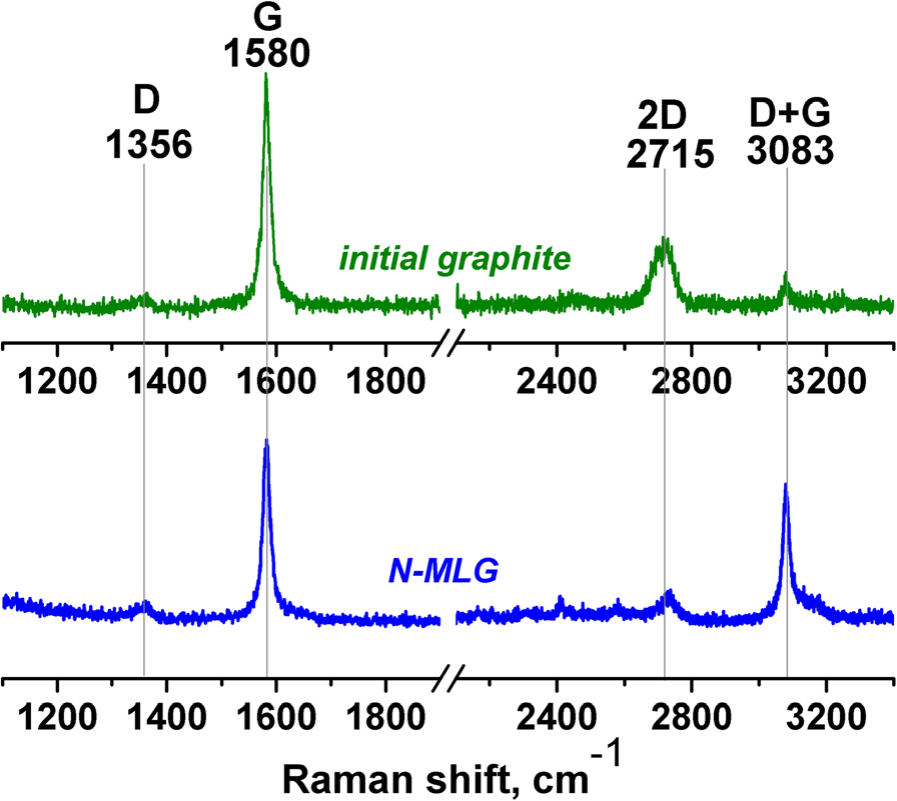
Raman spectra of initial graphite and N-MLG

Raman spectrum of obtained N-MLG differs from spectrum of starting graphite, and it is characterized by typical for carbon materials D, G, and 2D bands (Fig. 5). The position of the 2D band at 2729 cm^−1^ along with the impossibility of its approximation via only one Lorentz line [27] as well as ratio I(G)/I(2D) >1 [28] indicate the multilayer nature of electrochemically produced N-graphene. It should be also mentioned the presence of intense enough D + G band in spectrum of N-MLG which may be attributed to N-modified graphenes [29].

**Fig. 6:**
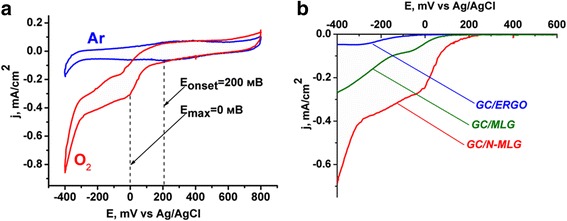
CVs of GC electrode modified with N-MLG in presence and absence of O_2_ (**a**) and electrocatalytic curves of GC electrode modified with ERGO, MLG, and N-MLG (**b**). Electrolyte—0.05 M H_2_SO_4_

As it is known, N-doping of graphene leads to significant improving of its functional characteristic in particular electrocatalytic activity in oxygen reduction reaction (ORR), which is current-forming process in fuel cells [30]. In order to evaluate activity of obtained N-MLG in ORR, the electrochemical characteristics of GC-electrodes, modified with N-MLG, in 0.05 M H_2_SO_4_ in presence and absence of oxygen were investigated by means of cyclic voltammetry. For comparison, the same features were measured for graphene materials, which did not contain nitrogen in their composition—electrochemically obtained multilayer graphene (MLG) [15] and electrochemically reduced GO (ERGO). As one can see from cyclic voltammograms (CVs) on Fig. 6, the usage of N-modified graphene instead of graphene materials without nitrogen allows to reduce ORR overpotential; it manifests in anodic shift of both onset potential (E_onset_) and catalytic current maxima potential. Improving of electrocatalytic properties of N-MLG in comparison with MLG and ERGO can also be an additional indirect proof of functionalization of obtained material by nitrogen atoms. It should also be noted that the electrocatalytic activity in ORR of obtained N-MLG is comparable with the established in the literature for the N-modified graphenes obtained by other methods [30].

**Fig. 7:**
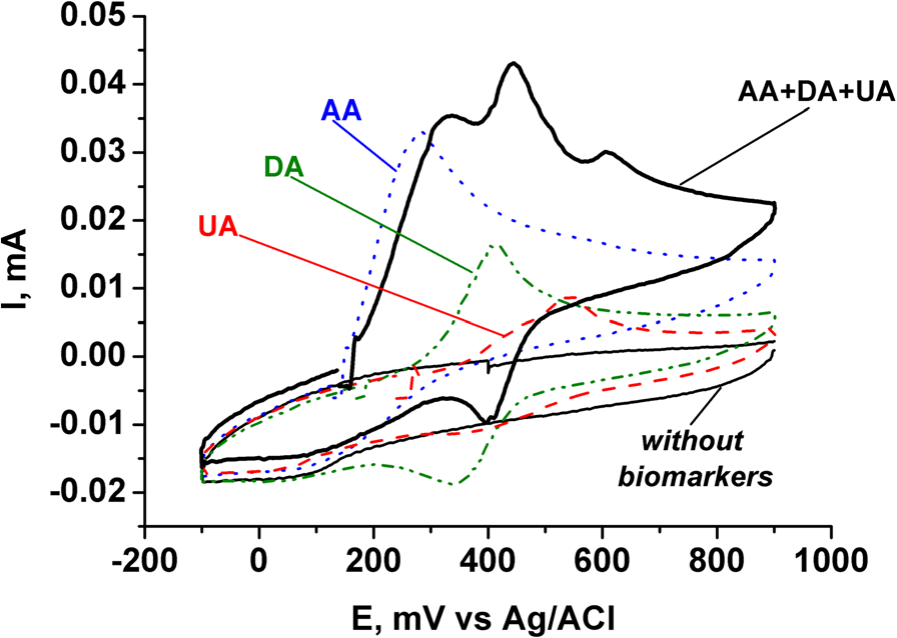
CVs of GC electrode modified with N-MLG in presence and absence of corresponding biomarkers (DA 1 mM, AA 5 mM, UA 8 mM) or their mixture in 0.1 M KCl

One important application of N-MLG may be its use as an electrode material for electrochemical analysis of biomarkers—dopamine (DA), ascorbic (AA), uric (UA) acids, etc., because doping graphene with heteroatoms may improve their sensitivity and selectivity at electrochemical determination of these substances. As a result of electrochemical studies, it was found that the biomarkers on glassy carbon (GC) electrode modified with N-MLG are oxidized at different potentials—AA 285 mV, DA 415 mV, and UA 535 mV (Fig. 7). It should be noted that when all three biomarkers are present in the electrolyte simultaneously, there are three separate peaks in CV, which obviously correspond sequential oxidation AA–DA–UA, while quite a significant difference between potentials of anodic peak maxima in CV (AA–DA ~120 mV, DA–UA ~165 mV) suggests the possibility of using N-MLG in electroanalysis of mentioned above biomarkers in their mixtures [31], for example, in biological liquids.

## Conclusions

In conclusion, the possibility of one-step electrochemical preparation of multilayer graphene functionalized with nitrogen (N-MLG) via exfoliation of graphite electrode in presence of azide anions in a pulse mode of electrolysis was presented. Sodium azide provides not only exfoliation of graphite via multiple repetition of anion intercalation/deintercalation cycles into graphite interlayer space but also simultaneous functionalization of graphene sheets by nitrogen atoms as a result of partial electrochemical decomposition of azide anions with ammonia evolution. Particles of N-MLG preferably consist of 7–9 individual graphene layers with a low amount of oxygen-containing defects (C/O ratio ~17), which was proved by means of C,H,N-analysis, TEM, AFM, FTIR, UV–Vis, and Raman spectroscopy. It was shown that increase of electrolyte concentration during electrochemical synthesis (from 0.1 to 2.0 M) allows change nitrogen content from 0.2 to 0.9% in resulting material.

It was found that N-MLG is a promising electrode material. By means of cyclic voltammetry, it was shown higher electrocatalytic activity of N-MLG in the oxygen reduction reaction, which is realized in fuel cells, compared to electrochemically prepared multilayer graphene or electrochemically reduced graphene oxide, that do not contain nitrogen atoms in their structure. Also, N-MLG was shown to be electrochemically active toward oxidation of such biomarkers as dopamine (DA), ascorbic (AA), and uric (UA) acids. Significant difference of oxidation potentials of DA, AA, and UA (when all three substances were present in the electrolyte simultaneously) suggests the possibility of using N-MLG in electroanalysis of mentioned above biomarkers in biological liquids.

## Abbreviations

AA: Ascorbic acid
AFM: Atomic force microscopy
CV: Cyclic voltammogram
DA: Dopamine
E_onset_: Onset potential
ERGO: Electrochemically reduced graphene oxide
FTIR: Fourier transform infrared spectroscopy
GC: Glassy carbon
GO: Graphene oxide
MLG: Electrochemically prepared multilayer graphene
N-graphene: Nitrogen-doped graphene
N-MLG: Multilayer nitrogen-doped graphene
ORR: Oxygen reduction reaction
TEM: Transmission electron microscopy
UA: Uric acid
